# The Gross Reproductive Anatomy of the Female Temminck's Pangolin *Smutsia temminckii* (Smuts, 1832)

**DOI:** 10.1111/ahe.70199

**Published:** 2026-11-11

**Authors:** Lauren Nicole Tink, Raymond Jansen, Christine Steyn

**Affiliations:** ^1^ Department of Nature Conservation, Faculty of Science Tshwane University of Technology Pretoria South Africa; ^2^ Department of Environmental, Water and Earth Science, Faculty of Science Tshwane University of Technology Pretoria South Africa; ^3^ IUCN SSC Pangolin Specialist Group c/o Zoological Society of London London UK; ^4^ Department of Anatomy and Physiology, Faculty of Veterinary Science University of Pretoria Onderstepoort South Africa

**Keywords:** bicornuate uterus, gross anatomy, ovaries, reproduction

## Abstract

Historically, pangolins have not been able to reproduce successfully in captivity and this may be in part due to the lack of knowledge and understanding with regards to the pangolin reproductive system (anatomy, physiology, biology) in all eight species. This original study describes the gross anatomy of the female Temminck's pangolin from three whole frozen adult individuals sourced from a registered biobank, in addition to four fresh female Temminck's pangolin reproductive tract specimens of varying size and weight obtained from a wildlife veterinary hospital following autopsy. The reproductive tract consists of a bicornuate uterus with convoluted uterine horns, similar to domestic ruminants. The ovaries are ovoid in shape and slightly bilaterally flattened. The difference in shape of the left ovary and uterine horn in relation to the right leads us to the possible conclusion that pregnancies are predominantly, although not exclusively, present in the left uterine horn of Temminck's pangolin. Furthermore, the bicornuate morphology of the uterus and uterine horns is attributed to the high maternal investment and energy requirement to produce a single, well‐developed offspring capable of locomotion soon after birth. This is a derived characteristic shared with ungulates. The well‐developed suspensory ligament suspends the ovaries and the uterus in the abdominal cavity. Both the predominance of pregnancies on the left side and the well‐developed suspensory apparatus of the reproductive tract may be an adaptation to the unique defensive characteristic of pangolins that allows them to roll up in a ball.

## 1 Introduction

The Temminck's pangolin (*Smutsia temminckii*) is one of eight pangolin species worldwide and is the only species present in southern Africa (du Toit et al. 2014; Pietersen et al. 2016; Pietersen et al. 2020). Pangolins are under threat worldwide due to illegal poaching (Pantel and Chin 2008; Challender et al. 2020), and all eight species have been classified on Appendix I of CITES (CITES 2022) and range from vulnerable (Temminck's pangolin and black‐bellied pangolin (*Phataginus tetradactyla*)) to critically endangered (the Chinese ( *Manis pentadactyla* ), Sunda ( *Manis javanica* ) and Philippine ( *Manis culionensis* ) pangolin) according to the IUCN (2019). An increased focus on the reproductive system can aid role‐players in better understanding and providing suitable management strategies for these endangered mammals.

To date there is little research published on the reproductive morphology of the female pangolin across the eight species. The literature that is available mostly pertains to reproductive parameters such as the gestational period (Chong et al. 2020; Mahmood et al. 2020; Wu et al. 2020), breeding seasons (Zhang et al. 2015; Chong et al. 2020) and age of sexual maturity (Chong et al. 2020; Gudehus et al. 2020). These parameters have either been studied only at a local level based on fragmentary reproductive records or are speculated based on singular observations due to the elusive nature of pangolins (Pietersen et al. 2020). Additionally, most of these parameters have been presented as a range and can vary widely within and between species (Jansen et al. 2020).

The Chinese pangolin has varying reports of gestational period. 180–225 days (Wu et al. 2020; Arora et al. 2023), 101–169 days (Hua et al. 2015; Sun et al. 2021) and 318–372 days (Chin et al. 2011) have been suggested. However, the speculated cause for these extreme differences has been attributed to potential delayed implantation, which warrants further investigation (Wu et al. 2020; Arora et al. 2023). Sexual maturity is reached as early as 6 months but generally by 1 year of age (Zhang et al. 2016; Wu et al. 2020). Zhang et al. (2015) report captive females becoming pregnant at a weight of 2 kg. The breeding season is between September and February after which one offspring is born (Thapa et al. 2014; Zhang et al. 2016; Wu et al. 2020); however, there have been rare reports of twin births (Wu et al. 2020).

Similarly, the Sunda pangolin shows varying reports on these reproductive parameters. The gestation period is estimated to be roughly 6 months (Zhang et al. 2017) with Zhang et al. (2015) and Chong et al. (2020) giving more specific estimates of 106–207 days and 168–188 days respectively. This species gives birth to one offspring per year (Lim and Ng 2008; Zhang et al. 2015; Zhang et al. 2017; Chong et al. 2020), with breeding suspected to occur all year round (Zhang et al. 2015; Chong et al. 2020).

With regards to the black‐bellied pangolin, gestation is estimated at 140 days after which a single offspring is birthed (Gudehus et al. 2020). Sexual maturity is thought to be reached by 2 years of age (Gudehus et al. 2020).

The white‐bellied pangolin (*Phataginus tricuspis*) has an estimated gestation period of 140–150 days; however, in captive scenarios this period has been observed to be as long as 8 months (Jansen et al. 2020). This variance is suspected to be as a result of embryonic diapause (Jansen et al. 2020). Focusing more on the physical reproductive anatomy, Ofusori (2007) investigated the morphostructure of the ovary of the white‐bellied pangolin and concluded that the left ovary may be the principal sex organ for reproduction in this species based on the consistent bilateral variation in size of the ovaries observed between ovaries.

Gaudin et al. (2020) describes the uterus of extant pangolin species as typically being bicornuate; however, the Chinese pangolin is described by Zhang et al. (2016) as having a bipartite uterus. Apart from these studies, the morphology of the female pangolin reproductive system is still largely unknown.

In the case of the Temminck's pangolin, there have been contradictory reports in some of these reproductive parameters in different geographical regions of southern Africa. It has been suggested by van Ee (1966) that the Temminck's pangolin produces a single offspring at birth (monotocous) and gives birth once a year; however, observations in the arid Kalahari region of northwest South Africa suggest that breeding may be biennial (Pietersen et al. 2020). Reports of the gestational period range from 105 to 140 days (Heath 1992; van Ee 1966; Swart 2013; Pietersen et al. 2020). Furthermore, the age of sexual maturity is thought to be in the range of 2 years, with reproduction commencing from 3 to 4 years once a home range has been established (Pietersen et al. 2014). However, more recent and consistent observations by the Johannesburg Wildlife Veterinary Hospital (JWVH) have suggested that the change from sub‐adult to adult occurs in the range of 6 kg in body weight (Pers. Comm. 2020).

The considerable variation in published reports and gaps in knowledge on these reproductive parameters across pangolin species illustrate, quite apparently, the need for more focused research into reproductive parameters.

## 2 Materials and Methods

Three female Temminck's pangolin of varying size and weight were obtained from the African Pangolin Working Group (APWG), a registered Non‐Profit Organization (NPO 123–147) (Pretoria, South Africa). These female pangolin carcasses had been recovered either after illegal poaching, and unfortunately succumbed to either disease or injuries associated with the illegal trade, or were collected after dying from electrocution. These individuals were frozen for research purposes at the SANBI Biobank (SANBI/RES/P2021/28). The frozen female pangolin carcasses were collected from SANBI and were processed at the Arcadia campus of Tshwane University of Technology in Pretoria. They were placed in 150 L containers and allowed to thaw and immersion‐fix in 10% neutral‐buffered formalin solution. Once partially thawed (24 h), a medial incision was made on the ventral surface from the base of the throat down to the pubis to ensure proper fixation and preservation of the internal organs. 10% neutral‐buffered formalin was also injected with a 20 *cc* syringe and 18‐gauge needle into large muscle areas of each carcass to ensure proper preservation. The pangolin carcasses were then left for a minimum of 2 weeks. Once completely preserved, one carcass was then removed at a time from the formalin and rinsed with running tap water for at least 48 h before the dissection commenced.

Four fresh female Temminck's pangolin reproductive tracts of varying size and weight were obtained from the JWVH (FH 17/15890) after the animals died due to disease or injuries related to the illegal trade. A full post‐mortem was performed on each of the animals (for legal purposes) by Dr. Sam Currie, registered veterinary pathologist at the Glens Veterinary Hospital (FHO97/0123) before the female reproductive tracts were removed and donated for this study. The reproductive tracts were placed in 10% neutral‐buffered formalin directly after the post‐mortems and were left for at least 1 week before examination. Two of the post‐mortems were observed by the author, and in both instances, a conceptus was observed in the left horn of the uterus.

Pangolins were named as follows: Females 1, 2, and 7 were from dissections; Females 3 and 4 were previous post‐mortems; and Females 5 and 6 were observed post‐mortems of pregnant females.

The dissections (Females 1, 2 and 7) were performed in a laboratory on the Arcadia campus of Tshwane University of Technology in Pretoria. Before dissections, the pangolin carcasses were weighed using a Rapala 25‐kg mini digital scale. Female pangolins that were below 6 kg were classified as sexually immature or juvenile, and those that exceeded 6 kg were classified as sexually mature or adult based on the extensive observations and experience of the JWVH (Pers. Comm. 2020).

Using the previous ventral medial incision, the layers of skin and abdominal muscles were bilaterally reflected laterally to gain better access to the abdominal and pelvic regions. The musculature of the female pangolin carcasses was quite rigid, potentially due to the stage of decay or rigour mortis when originally frozen, and a basic retractor was used to assist in holding the hind legs apart. The pubis was excised using short nose side cutters to expose the reproductive tract. The reproductive tract (organs and ligament attachments) was observed and photographed by one of the authors, LNT (iPhone 8, resolution at 12 Megapixels) in situ before being removed in its entirety and placed in 10% neutral‐buffered formalin. Prior to further analysis the reproductive tract was rinsed for at least 48 h.

*Ex situ* observations of Females 1–7: photographs and measurements using a digital vernier calliper (Dexter 0–150 mm) were taken. The uterus, vagina and vaginal vestibule were dissected dorsally in order to further investigate and describe internal structures. Each measurement in the study was taken three times and an average calculated for the final measurement.

The following measurements were taken:

Both the left and right ovary were measured from the cranial pole to the caudal pole (length), and from the mesovarial margin to the free margin (width) at the widest point. The uterine body measurement was taken from the point of convergence of the uterine horns to the most caudal end of the *Portio vaginalis*. The vagina was measured from the cranial *vaginal fornix* to the transverse fold. Accurate measurements of the uterine horns and the uterine tubes could not be obtained due to the convoluting anatomic configuration.

In addition to these measurements, nine (*n* = 9) computed tomography (CT) scans performed at Blue Hills Veterinary Hospital (Midrand, Johannesburg) of previous pregnant pangolin patients of the APWG were assessed by one of the authors, LNT, in order to determine the number of left versus right horn pregnancies.

This was a descriptive study and data was presented as a range. Due to the sensitive nature of the study species, the sample size was not appropriate for statistical analyses.

The terminology used in this study follows that of *Nomina Anatomica Veterinaria* 5th Edition (Revised) (ICVGAN 2012).

## 3 Results

Due to the association of the urogenital tract with the reproductive tract, a basic description of the topography of the kidneys was included. The kidneys were positioned dorso‐medially in the abdominal cavity, with the left kidney presenting more caudal and the right kidney more cranial (Figure 1).

**FIGURE 1:**
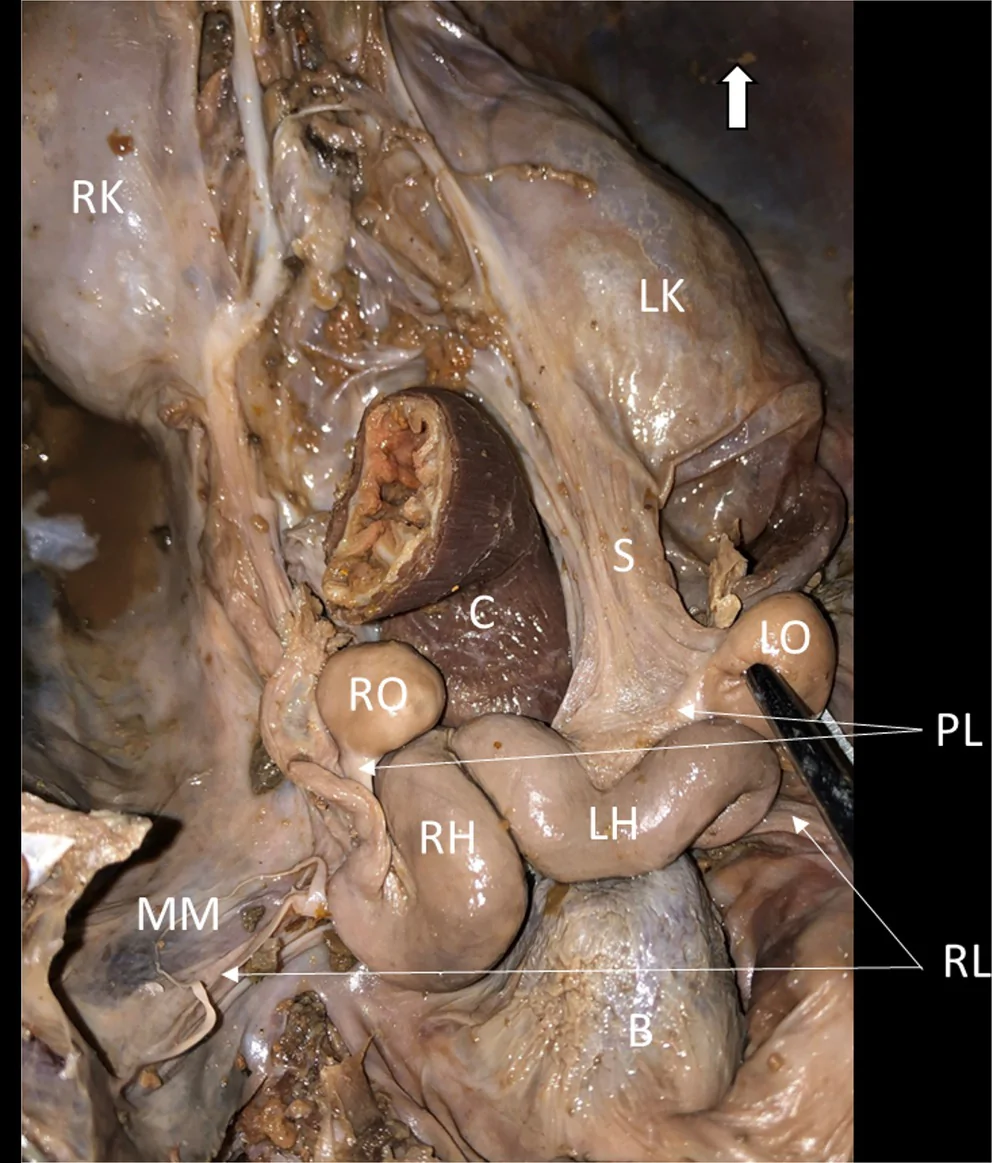
Ventral view of the caudal abdomen of a 10.52 kg adult female Temminck's pangolin, cranial portion of the large colon transected and removed. Bladder (B). Colon (C). Left uterine horn (LH). Left Kidney (LK). Left Ovary (LO). Mesometrium (MM). Proper Ligament of the ovary (PL). Right uterine horn (RH). Right kidney (RK). Round Ligament (RL). Right Ovary (RO). Suspensory Ligament (S), arrow indicates cranial direction.

The sample set consisted of individuals of varying sizes and weights, and some samples without body weight recorded (post‐mortem) (Table 1). However, for these data deficient samples (Females 3 and 4), it was inferred that these females were not sexually mature adults and were nulliparous (not having given birth), based on the following observations: all the measurements from Females 3 and 4, with the exception of the length of the right ovary, were smaller than that of Female 1. Since Female 1 weighed 5.5 kg, which is close to the threshold for sexual maturity (as observed by JWVH), it was inferred that Females 3 and 4 weighed less than Female 1, and therefore were not sexually mature. In addition, the uterine horns of Females 3 and 4 were most convoluted, and the broad ligaments were not elongated, indicating that they most likely had not been pregnant previously.

**TABLE 1:** Measurements (mm) of female reproductive tracts.

Female	Left ovary		Right ovary		Uterine body	Cervix	Vagina	Vestibule	Body weight (kg)
	Width	Length	Width	Length					
1	7.45	13.87	7.39	12.29	36.48	5.36	27.67	15.59	5.5
2	14.02	11.91	11.54	12.78	42.56	5.95	38.12	—	—
3	6.61	8.35	6.20	7.14	14.6	3.41	12.36	—	—
4	6.78	12.6	7.63	14.96	17.39	—	—	—	—
5	13.49	22.06	10.34	21.73	71.39	8.54	39.19	22.91	10.25
6	8.89	13.88	7.90	11.86	58.42	6.90	—	—	8.25
7	11.42	13.94	9.19	13.74	42.79	8.03	38.92	23.46	11.02

### 3.1 Ovary

The ovaries were ovoid in shape and slightly bilaterally flattened (Figure 2). Female 2 (sub‐adult) presented slightly pointed poles; however, still conformed to the ovoid shape. Each ovary was positioned caudal to and attached to the ipsilateral kidney by the suspensory ligament (see Section 3.4) with the cranial pole (*Extremitas tubaria*) of the ovary positioned craniolaterally and the caudal pole (*Extremitas uterina*) caudomedially (positioned at an angle). The free margin (*Margo liber*) was directed medially, while the mesovarial margin (*Margo mesovaricus*) was directed laterally and attached by means of the proper ligament of the ovary (see Section 3.4) to the uterine horn. The measurements of the ovaries indicated that the left ovary was larger than the right (both in width and length), with the exception of Female 4 and the length of the ovary in Female 2 (Table 1). The left ovaries were 8.35–22.06 mm in length and 6.61–14.02 mm in width. The right ovaries measured 7.14–21.73 mm in length and 6.20–11.54 mm in width.

**FIGURE 2:**
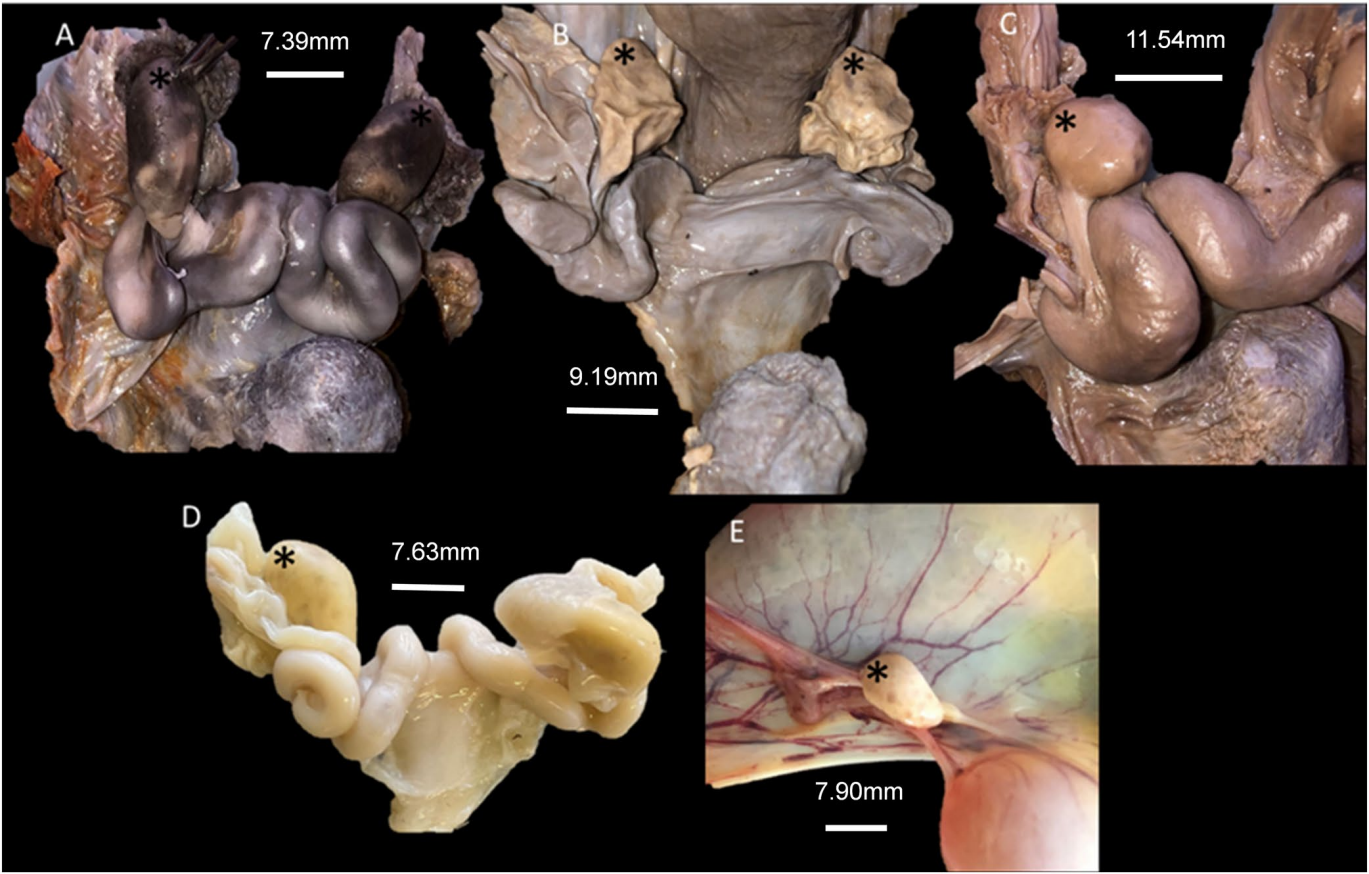
Ventral view of the ovaries and uterine horns of Temminck's pangolin Female 1 (A); 7 (B); 2 (C); 4 (D); 6 (E). *Indicates cranial pole orientation.

There were several *Corpora lutea* present on the ovaries of the pregnant females (Female 5 and Female 6), giving the ovaries a pitted appearance (Figure 3). No follicles were visible on the ovaries of the remaining samples.

**FIGURE 3:**
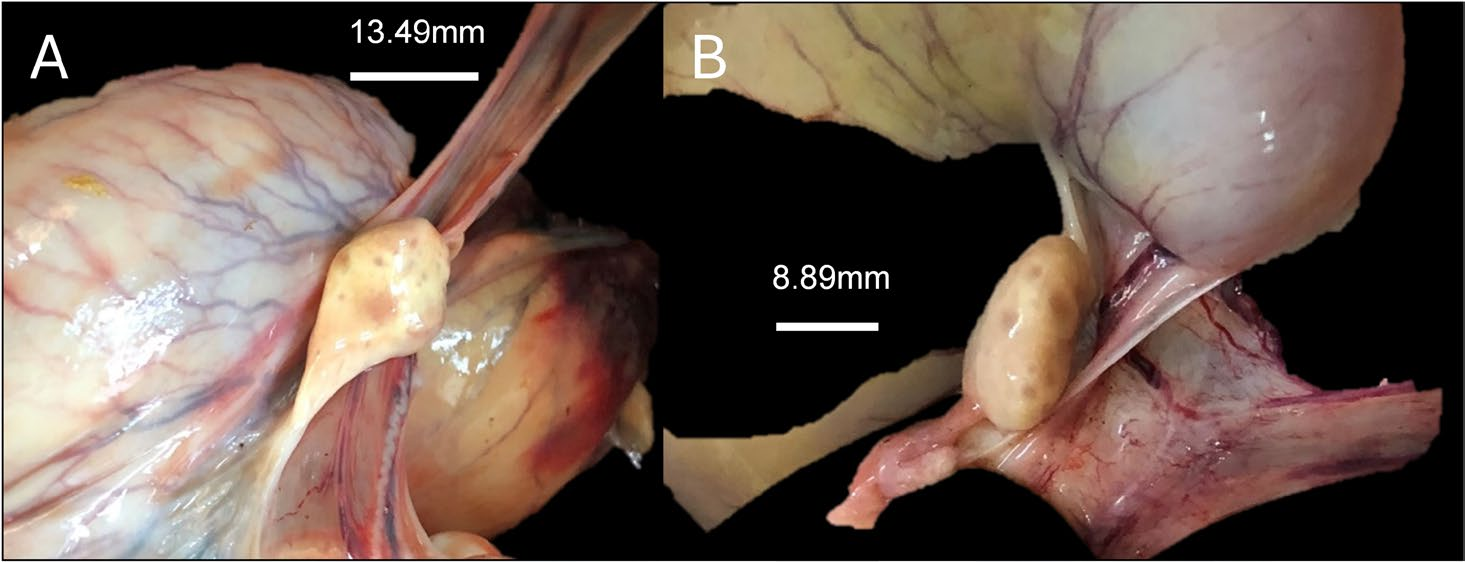
Ventral view of ovaries of pregnant Female 5 (A) and 6 (B) showing *Corpora lutea*.

Female 7 presented ovaries that appeared to have a decreased volume and a shrivelled appearance. This was also the case for the uterine horns and uterine body of this specimen (Figure 2B).

### 3.2 Uterus and Uterine Tube

The shape of the uterus was consistent with the description of a bicornuate form (*Uterus bicornis*) as it consisted of two well‐developed uterine horns (*Cornu uteri*) that merge into the uterine body (*Corpus uteri*) without an internal septum. The uterine horns were convoluted more so than that of a bovine but less than that of an ovine. The uterine horns diverged cranially from the uterine body and tapered as they continued into the convoluted uterine tubes (*Tuba uterina*) and terminated in a well‐developed infundibulum (*Infundibulum tubae uterinae*) and fimbriae (*Fimbriae tubae*) that extended over the length of the ovary (Figure 4C). The free margin of each uterine horn was positioned medially while the mesovarial margin was positioned laterally and attached to the lateral body wall via the broad ligament (see Section 3.4) (Figure 5). The left uterine horn was consistently more stretched out/less convoluted than the right uterine horn.

**FIGURE 4:**
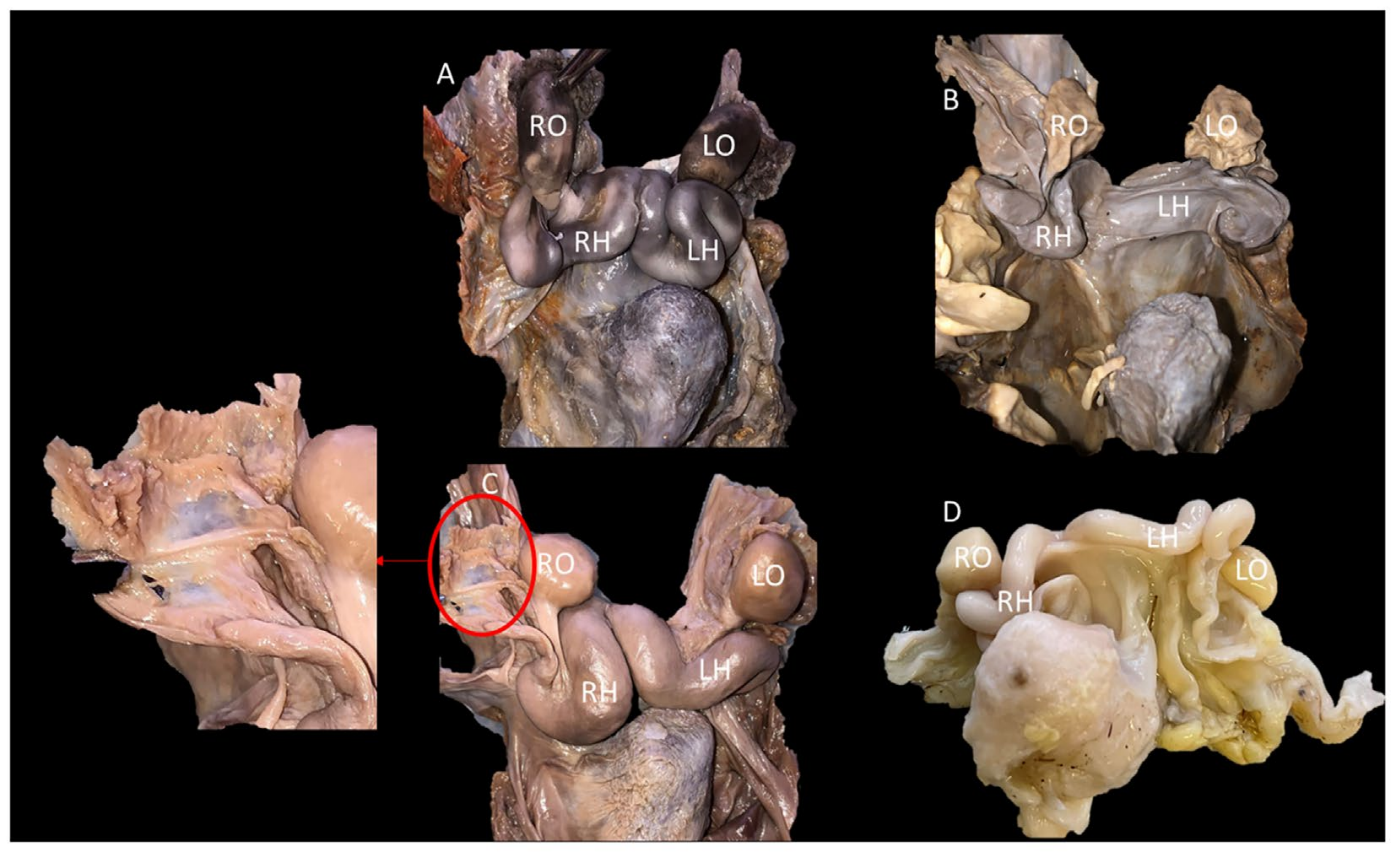
Ventral view of the uterine horns of Temminck's' pangolin Female 1 (A); 7 (B); 2 (C); 4 (D). Left horn (LH). Left ovary (LO). Right horn (RH). Right ovary (RO). The red circle indicates zoomed‐in view of the infundibulum and fimbriae of Female 2.

**FIGURE 5:**
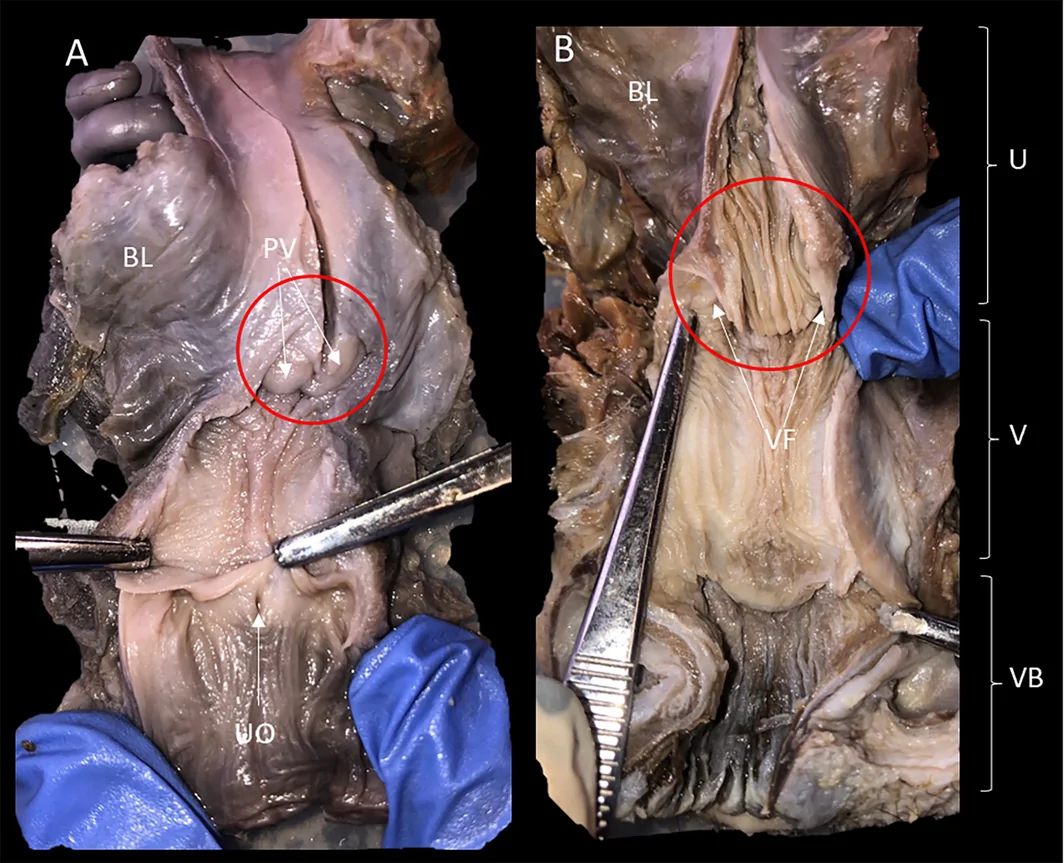
Ventral view of the uterine body, vagina and vaginal vestibule of the Temmincks' pangolin Female 7, weight 11.02 kg. (A) *Portio vaginalis* (PV); Urethral opening (UO). (B) Uterus (U). Vagina (V). Vaginal Vestibule (VB). Vaginal Fornix (VF).

A well‐developed cervix (*Cervix uteri*) was positioned at the caudal end of the uterine body and consisted of a cervical part (*Portio prevaginalis*) cranially and a vaginal part (*Portio vaginalis*) caudally. The lumen of the cervix, the cervical canal (*Canalis cervicis uteri*), presented well‐developed longitudinal folds and opened cranially into the uterine body at the internal uterine opening (*Ostium uteri internum*) and caudally into the external uterine opening (*Ostium uteri externum*). The most caudal part of the cervix, the *Portio vaginalis*, projected into the vaginal lumen, and the vaginal fornix (*Fornix vaginae*) (see Section 3.3) formed an anular ring around it. Both the *Portio vaginalis* and the vaginal fornix were quite prominent. Female 5 was pregnant, and a mucus plug was observed in the cervical region.

### 3.3 Vagina and Vaginal Vestibule

The vagina extended from the external opening of the uterus to a prominent transverse fold on the vaginal floor. The most cranial part of the vagina forms the vaginal fornix, a blind‐ending ring‐like space around the *Portio vaginalis* of the cervix. The external urethral opening (*Ostium urethrae externum*) was located ventral to the prominent transverse fold and opened in the area caudal to the transverse fold termed the vaginal vestibule (*Vestibulum vaginae*). Longitudinal folds were present in both the vaginal canal and the vaginal vestibule, although these appeared to be more pronounced in the vaginal vestibule (Figure 5). In addition, smaller transverse folds were observed at both the cranial and caudal parts of the vaginal canal.

The tubular parts (uterus, vagina, and vestibule) occupied a medial position in the pelvic cavity.

### 3.4 Suspensory Apparatus of the Reproductive Tract

The reproductive tract was supported and attached to the dorsal and lateral walls of the abdominal cavity by means of a series of ligaments, collectively termed the broad ligament (*Ligamentum latum uteri*), comprising the *Mesovarium*, *Mesosalpinx*, and *Mesometrium*. Further supportive ligaments were the suspensory ligament (*Ligamentum suspensorium ovarii*), round ligament (*Ligamentum teres uteri*), and the proper ligament of the ovary (*Ligamentum ovarii proprium*).

The well‐developed suspensory ligament originated in a broad strap‐like manner caudomedially from the dorsal side of the kidney and attached to the mesovarial margin of the ovary (Figure 1). This then continued caudally and attached to the most lateral bend in the uterine horn as the mesometrium. Furthermore, the mesometrium extended transversely to attach to the lateral body wall. From the mesovarium a fold originated that suspends the uterine tubes termed the Mesosalpinx (Figure 6).

**FIGURE 6:**
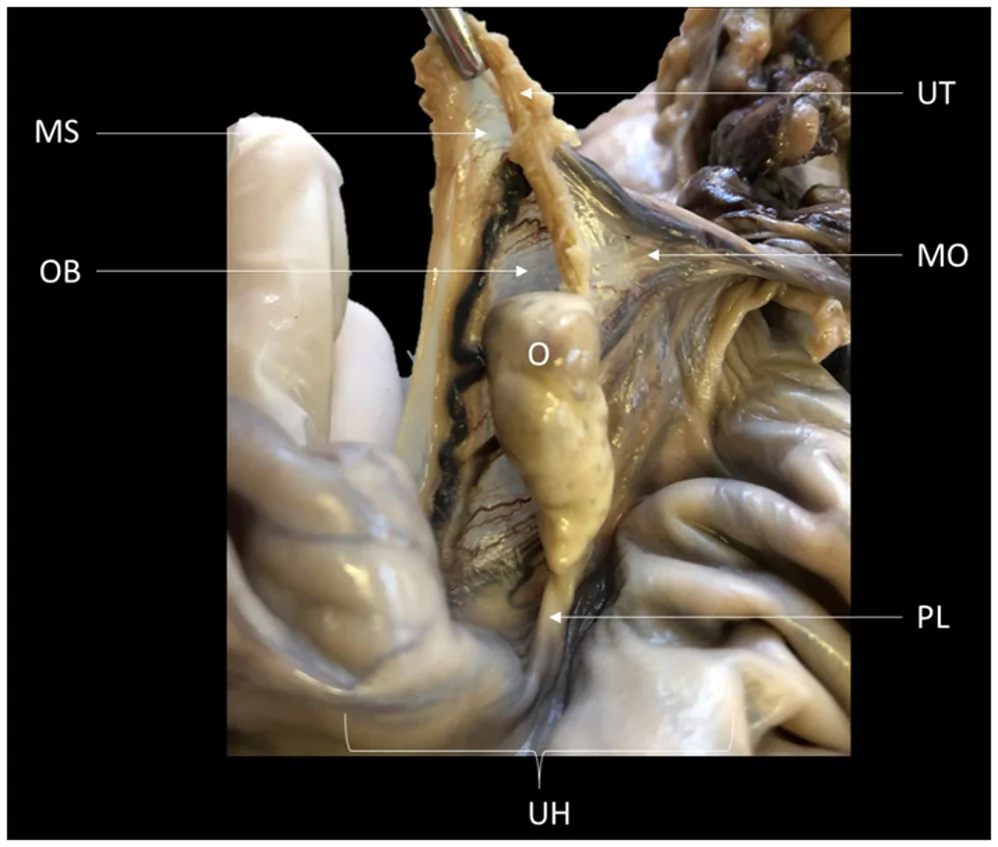
Ventral view of Female 5 (weight 10.20 kg) indicating the Mesovarium (MO), Mesosalpinx (MS), Ovary (O), Ovarian bursa (OB), Proper ligament of the ovary (PL), Uterine horn (UH), Uterine tube (UT).

The round ligament arose as a fold from the lateral surface of the caudal section of the mesometrium and was quite well developed (Figure 7). Its insertion was in the inguinal area.

**FIGURE 7:**
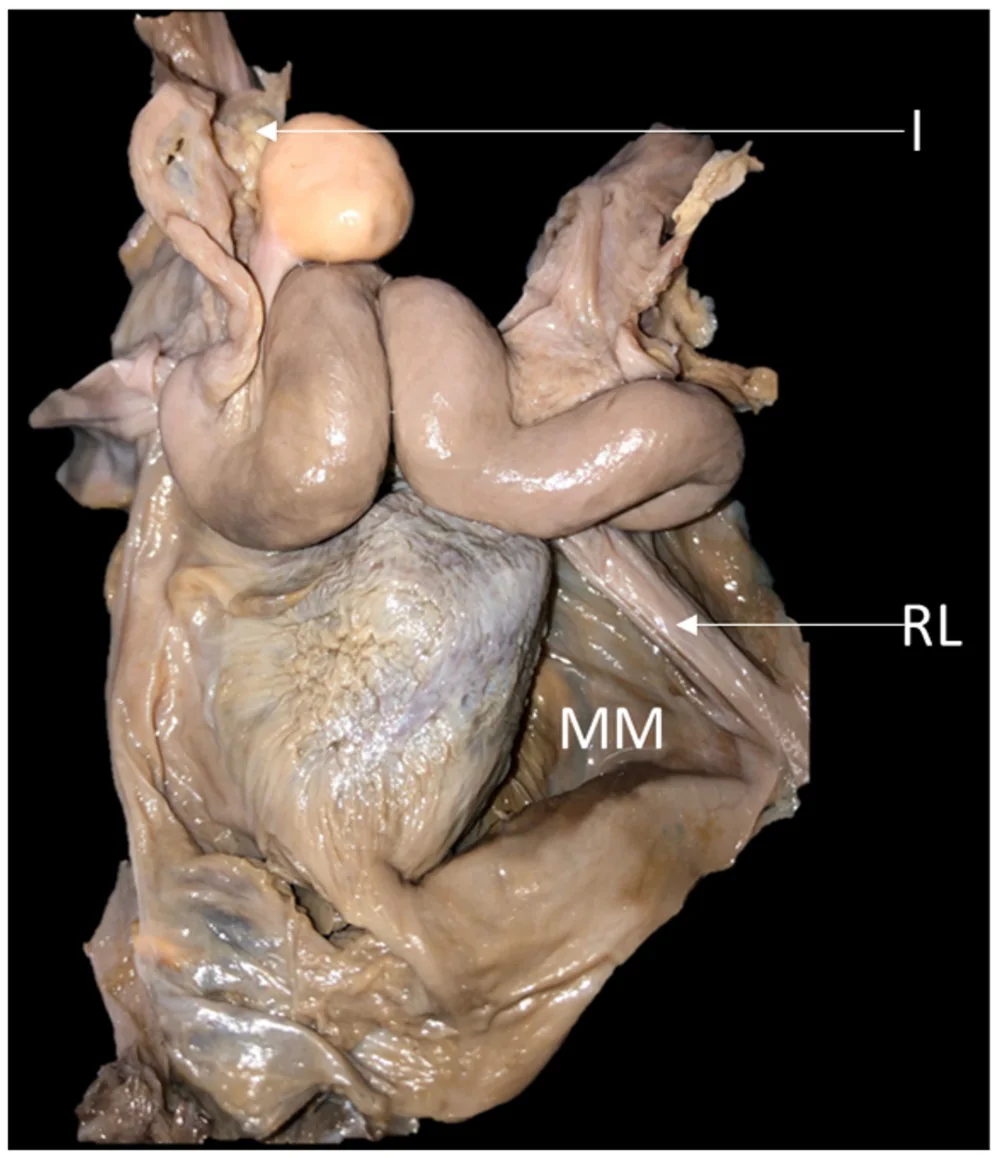
Ventral view of Female 2 (weight 10.50 kg) indicating the Infundibulum (I). Mesometrium (MM). Round ligament (RL).

The cranial end of each uterine horn was attached to the ovary by the proper ligament of the ovary. This was a short broad ligament (Figures 1 and 6).

The Mesosalpinx and mesovarium formed a small pouch, termed the ovarian bursa. This pouch was reduced and did not enclose the ovary (Figure 6).

At the divergence of the uterine horns, there was a small but poorly developed intercornual ligament (*Ligamentum intercornuale*) present.

### 3.5 Observations of CT Scans

Of the nine pregnancies that were observed on the CT scans, eight (*n* = 8) pregnancies were identified in the left uterine horn, and only one (*n* = 1) in the right uterine horn.

## 4 Discussion

Pangolins are taxonomically unique and because of this, the challenge lies in selecting an appropriate reference species when describing and discussing the reproductive morphology of the Temminck's pangolin.

### 4.1 Species Potentially Similar due to Convergence

Pangolins were long thought to be closely related to the Xenarthra (anteaters, tree sloths and armadillos) (Rose and Gaudin 2010; Gaubert et al. 2018). This was based on their shared derived characteristics, primarily their adaptations for digging and for feeding on ants and termites. However, this is ultimately attributed to convergent evolution rather than close genetic similarity (Rose and Gaudin 2010; Gaubert et al. 2018).

The order Xenarthra is a morphologically diverse group (Rossi et al. 2011). Within the Xenarthrans, the nine‐banded armadillo ( *Dasypus novemcinctus* ) has an annual breeding season with a gestational period of 4–5 months and is capable of the phenomenon of specific polyembryony, which is the formation of more than one embryo from a single fertilized ovum (Talmage and Buchanan 1954; Loughry et al. 1998). Their ovaries are bean‐shaped and located caudal and lateral to the kidneys, with a simplex uterus (simple, pear‐shaped form) similar to that of primates (Newman and Patterson 1910; Talmage and Buchanan 1954; Fromme et al. 2021). A simplex uterus typically possesses a single uterine body and cervix without noticeable uterine horns (Machado et al. 2022). For a species classed as phylogenetically primitive, the structure of the reproductive tract contains a mixture of both archaic and specialized characteristics (Talmage and Buchanan 1954). Other species of armadillo such as the southern long‐nosed armadillo ( *Dasypus hybridus* ), Brazilian three‐banded armadillo ( *Tolypeutes tricinctus* ), and the southern three‐banded armadillo ( *Tolypeutes matacus* ) all possess a simplex uterus, whereas species such as the big hairy armadillo ( *Chaetophractus villosus* ) and the pygmy armadillo ( *Zaedyus pichiy* ) possess a more primitive bicornuate uterus (Talmage and Buchanan 1954).

Both the lesser anteater ( *Tamandua tetradactyla* ) and the greater anteater ( *Myrmecophaga tridactyla* ) possess a pear‐shaped simple uterus, a urogenital sinus as opposed to a separate vaginal canal, and a poorly developed cervix (Rossi et al. 2011; Fromme et al. 2021). The ovaries are bilaterally flattened and ovoid in shape (Rossi et al. 2011; Fromme et al. 2021). The greater anteater is a nonseasonal breeder with a gestational period of 180–190 days, in which one offspring is born (Patzl et al. 1998).

The sloths are comprised of two genera, *Bradypus* (three‐toed sloths) and *Choloepus* (two‐toed sloths). Information on the reproductive parameters of these species is sparse, however *Bradypus* has been described more than *Choloepus* with regards to the reproductive anatomy. The brown‐throated sloth ( *Bradypus variegatus* ) and pale‐throated sloth ( *Bradypus tridactylus* ) have oval‐shaped ovaries and a simplex/simple pear‐shaped uterus (Wislocki 1928; Favoretto et al. 2015), which is connected to a urogenital sinus by double cervices (Wislocki 1928; Favoretto et al. 2015). The brown‐throated sloth is reported to have a gestation period of between 4 months (Wislocki 1928) and up to 6 months (Taube et al. 2001; Hayssen 2010). Taube et al. (2001) describes the species as having a breeding season, however this varied dependant on climatic differences and between years of observations. The pale‐throated sloth has an estimated gestation period of between 106 days and 6 months (Hayssen 2009). This species has a distinct breeding season and reaches sexual maturity at 3 years (Taube et al. 2001).

The Hoffmann's two‐toed sloth ( *Choloepus hoffmanni* ) and the Linnaeus's two‐toed sloth ( *Choloepus didactylus* ) are both described as having a simplex/simple pear‐shaped uterus (Wislocki 1928; Hayssen 2011). The Hoffmann's two‐toed sloth has a gestation period of 11–12 months (Taube et al. 2001), and the Linnaeus's two‐toed sloth has a gestation period varying between 5 and 10 months (Taube et al. 2001), with ages of sexual maturity being 2–4 years and 3–4 years, respectively (Taube et al. 2001; Hayssen 2011).

There is a noted difference between the uterus shape of Xenarthra (predominantly *simplex uterus*) compared to pangolin species. As mentioned before, Gaudin et al. (2020) describe the uterus of extant pangolin species as typically being bicornuate–two uterine horns, a single uterine body and a single cervix (Machado et al. 2022). This type of uterus is present in mammals such as canines, felines, equines, porcines and ruminants (Machado et al. 2022). Zhang et al. (2016) describe the Chinese pangolin as having a bipartite uterus, but this is a term generally used to refer to a uterus with two uterine horns that connect directly to a cervix (and therefore the absence of a uterine body) (Machado et al. 2022). This type of uterus is usually present in rodents such as mice and rats (Machado et al. 2022). This study also confirms the bicornuate shape of the uterus in Temminck's pangolin.

### 4.2 Species Potentially Similar due to Geographic Similarity

The aardvark ( *Orycteropus afer* ) is the only extant species within its order Tubulidentata. This species occupies a similar geographic region and ecological niche to the Temminck's pangolin. With its nocturnal and fossorial habits as well as myrmecophagous diet, one could easily see the plausibility of drawing a comparison to the Temminck's pangolin. As with pangolins, the aardvark has not been widely studied in terms of its reproductive anatomy. Knöthig (2005) described the uterus as being primitive and resemblant of the duplex uterus, which typically possesses two separate uterine horns, each with a cervix (Machado et al. 2022). The gestation period is estimated to be 243 days (Parys et al. 2012). The morphology of the uterus of the aardvark differs greatly from that of the Temminck's pangolin and is therefore probably not a suitable species to use in comparison.

### 4.3 Species Potentially Similar due to Genetic Similarity

Phylogenetically, it has been proven that the closest genetic relative of the pangolin is the order Carnivora (Murphy et al. 2001; Gaudin et al. 2009; du Toit et al. 2014). Within Carnivora is the Aardwolf ( *Proteles cristata* ), a unique, myrmecophagous species that again fills a similar ecological and geographical niche to that of the Temminck's pangolin. With the majority of information available being based on behavioural research, not much is known of their reproductive anatomy or parameters other than this species being seasonal breeders (Marneweck 2013).

Due to the general lack of reproductive knowledge and the large variation in reproductive parameters amongst the species similar to the Temminck's pangolin based on convergence (Xenarthra), the comparative literature used was that of a genetically similar species (Carnivora). In general, there are few anatomical studies on wild carnivores, specifically focusing on the reproductive tract anatomy; however, Hartman (2012) comprehensively describes the reproductive anatomy of the African lion ( *Panthera leo* ). One notable recorded difference between pangolins and carnivores is that pangolins are recorded as being monotocous (producing a single offspring at a birth) and rarely having twins in some Asian species (Chong et al. 2020; Pietersen et al. 2020; Mahmood et al. 2020; Wu et al. 2020), whereas carnivores are classified as being polytocous, having two or more offspring at a time (Frandson and Spurgeon 1992; Faya et al. 2011; Holt et al. 2014).

### 4.4 The Reproductive Tract

In the Temminck's pangolin, the ovaries are ovoid in shape with some slight shape variation observed between samples. The ovaries of Females 1, 3, and 4 were more pointed at both poles but still conformed to the ovoid shape. This could be attributed to sexual immaturity, as these three samples were considered to be sexually immature individuals based on body weight being less than 6 kg (Female 1) (Table 1), and the physical size of reproductive organs in relation to the known samples as Females 3 and 4 were obtained from previous post‐mortems, and no record of body weight was available. It is assumed that these females were also nulliparous (not having given birth) (Females 3 and 4). The shape of the ovary is influenced by whether the individual is a juvenile, reproductively active, or senile (Talmage and Buchanan 1954; Chatdarong 2003). The ovaries of Females 2, 5, and 6 conformed to a typical ovoid shape. Females 5 and 6 were both pregnant at the time of dissection. Female 2 was classified as sexually mature based on body weight exceeding 6 kg (Table 1).

The pregnant females presented obvious *Corpora lutea* on the ovaries. There were no obvious follicles visible on the ovaries of the remaining samples. This could be expected as Females 1, 3 and 4 were classified as sexually immature. Female 7 was classified as being senile and the deflated appearance of the ovaries obscured the visibility of any *Corpus luteum* present. It would be expected to see follicles or *Corpus lutea* on the ovaries of Female 2 as this sample was classified as sexually mature. However, it is possible that because the sample was not fresh and was originally frozen and defrosted, some discolouration of the tissue could obscure the visibility of follicles or *Corpus lutea*.

The left ovary of all but Female 4 was larger than the right ovary. This was consistent with the finding of Ofusori (2007) that the left ovary was the principal sex organ for reproduction in the white‐bellied pangolin ( *P. tricuspis* ) based on the left ovary being consistently larger. This is also consistent with Hartman (2012) finding that the left ovary of the lion is longer and wider than the right ovary. Although CT scans from other pangolin patients at the APWG indicated that pregnancies were carried in both the left (*n* = 8) and right (*n* = 1) uterine horns, the rest of the findings in this study, as well as those of previous literature, indicate that pregnancies are predominant in the left uterine horn.

Associated with the ovary is an ovarian bursa, the pouch formed where the Mesosalpinx and mesovarium merge. In the Temminck's pangolin, the ovarian bursa is very shallow and does not entirely envelop the ovary. This has also been described in lions (Hartman 2012). The uterine body of the Temminck's' pangolin is short with relatively long and broad convoluted uterine horns with the caudal and cranial end lying close together. The pelvis is narrow and vertically orientated to support weight bearing bipedal locomotion (Hoffmann et al. 2020). The ovaries are located near to the cranial end of the uterine horn, almost immediately before the pelvis, with a long suspensory ligament attaching caudomedially to the dorsal side of the kidney. This differs from the general carnivoran that has a short uterine body with long slender uterine horns and ovaries supported by a short suspensory ligament (Dyce et al. 1996; Hartman 2012). Furthermore, the anatomical difference in the uterus and uterine horns can be attributed to the difference in the number of offspring between monotocous pangolin and polytocous carnivoran species. The uterus and uterine horns of the pangolin appear more similar to that of ungulates rather than that of carnivorans (Frandson and Spurgeon 1992; Dyce et al. 1996). Pangolins and ungulates exhibit shared derived characteristics of the uterus shape as well as the placentation of the foetal membrane (Carter and Mess 2017). This can be linked to the heavy maternal investment and energy requirement of “prey species” in pregnancy to produce one well developed offspring capable of locomotion soon after birth. Pangolins are documented to give birth to a single, relatively well‐developed offspring that is able to cling to and ride on the mothers back until it is able to forage for itself (Mahmood et al. 2020; Wu et al. 2020).

Female 2 was thought to have previously given birth due to the very apparent asymmetry of the broad ligament being observed. The left side was noticeably more stretched; additionally, the left uterine horn was more stretched/less convoluted than the right. It was thought that a previous pregnancy may have resulted in the straightening asymmetry of the uterine horns. This left‐sided straightening asymmetry of the uterine horn was consistently observed in the sexually mature females (Females 2 and 7).

Female 7 was the only distinctly different sample. The reproductive organs were notably reduced in volume and had a shrivelled or atrophied appearance, suggesting the possibility of anestrus. During anestrus, the dormancy of the ovaries and associated hormonal deprivation to the reproductive organs result in the atrophy of these organs (Frandson and Spurgeon 1992). Upon external examination, this female had a high tick (Order: Ixodida) parasite load in between scales and on the exposed underparts. This could lead to possible anaemia and poor body condition due to tick‐borne diseases (Frandson and Spurgeon 1992). Additionally, the scales were very thick and worn down with a smoothed edge, indicative of an aged animal (Pietersen et al. 2020). Therefore, the anestrus and associated atrophy could be explained as a result of advanced age, which could be considered senile and past the age for sexual activity.

The asymmetrical appearance and orientation of the reproductive organs and associated ligaments in the Temminck's pangolin are possibly attributed to previous pregnancies, but are more likely attributed to the unique tongue structure of this species. The tongue structure of the white‐bellied and giant pangolin was investigated (Doran and Allbrook 1973) and found to be positioned ventral to the larynx and trachea, running into the abdomen with the caudal portion located in the right iliac fossa, curving dorsolaterally to terminate in a spatula‐like shape in the region of the right crus of the diaphragm and the abdomen.

This space occupied on the right side of the abdomen would then potentially restrict the space available for the right uterine horn, resulting in the more convoluted appearance when compared to the left uterine horn. The space‐occupying structure may also be why more pregnancies are noted on the left side of the reproductive tract compared to the right side. As both horns have been noted to carry pregnancies in Temminck's pangolins, we are therefore not of the opinion that the left ovary is the primary sex organ for reproduction, but rather that pregnancies are more likely to occur in the left horn.

As pangolins adopt a defensive posture by ventral flexion when under attack, this may suggest that a lot of movement of the gravid uterus will take place in a pregnant female, consistent with what was observed in other African pangolin species (Doran and Allbrook 1973). The well‐developed suspensory apparatus in this species will aid the gravid uterus in movement and returning to its original position.

## 5 Conclusion

Temminck's pangolin is a unique species, and the reproductive tract of the female is no exception. The reproductive tract consists of a bicornuate uterus with convoluted uterine horns, similar to domestic ruminants. The ovaries are ovoid in shape and slightly bilaterally flattened. The difference in shape of the left ovary and uterine horn in relation to the right leads us to the possible conclusion that pregnancies are predominantly, although not exclusively, present in the left uterine horn of Temminck's pangolin. Furthermore, the bicornuate morphology of the uterus and uterine horns is attributed to the high maternal investment and energy requirement to produce a single, well‐developed offspring capable of locomotion soon after birth. This is a derived characteristic shared with ungulates. The well‐developed suspensory ligament suspends the ovaries and the uterus in the abdominal cavity. Both the predominance of pregnancies on the left side and the well‐developed suspensory apparatus of the reproductive tract may be an adaptation to the unique defensive characteristic of pangolins that allows them to roll up in a ball.

The vulnerable status of the Temminck's pangolin indicates the dire need to understand this mammal. Describing and understanding the reproductive tract is the crucial first step in understanding the reproductive biology in its entirety. This research has the potential to inform future captive breeding research and the implementation of conservation planning measures to save these species, as the need for these programs becomes more apparent with increasing poaching pressures.

## Funding

All funding to perform the anatomical dissection was made available by Tshwane University of Technology. Funding to support LNT towards her academic qualification at the University was graciously supported by the African Pangolin Working Group.

## Ethics Statement

Ethical clearance for this study was submitted to and approved by the Tshwane University of Technology's Animal Research Ethics Committee through the Faculty Research and Innovation Committee (Ref #: AREC2020/06/002). Government research approval from the Department of Agriculture, Land Reform and Rural Development in terms of Section 20 of the Animal Diseases Act (35 of 1984) was approved and granted valid from 15 February 2022 to 20 August 2024. A SANBI National Zoological Gardens Animal Research Ethics and Scientific Committee (ARESC) approval was granted (SANBI/RES/P2021/28). Furthermore, all legal documentation to receive, possess, transport, and dissect dead pangolins was applied for and obtained from the National Department of Forestry Fisheries and Environment (Original Permit number 278/64, first renewal permit number 27875, second renewal permit number 52917).

## Consent

The authors have nothing to report.

## Conflicts of Interest

The authors declare no conflicts of interest.

## Data Availability

The data that support the findings of this study are available from the corresponding author upon reasonable request.
